# Bayesian spatio-temporal modelling of tuberculosis in Vietnam: Insights from a local-area analysis

**DOI:** 10.1017/S0950268825000214

**Published:** 2025-02-12

**Authors:** Long Viet Bui, Romain Ragonnet, Angus E. Hughes, Hoa Binh Nguyen, Nam Hoang Do, Emma S. McBryde, Justin Sexton, Thuy Phuong Nguyen, David S. Shipman, Greg J. Fox, James M. Trauer

**Affiliations:** 1 School of Public Health and Preventive Medicine, Monash University, Melbourne, VIC, Australia; 2 National Tuberculosis Program, Hanoi, Vietnam; 3 National Lung Hospital, Hanoi, Vietnam; 4 Australian Institute of Tropical Health & Medicine, James Cook University, Townsville, QLD, Australia; 5 Commonwealth Scientific and Industrial Research Organisation (CSIRO), Canberra, ACT, Australia; 6 Sydney Medical School, University of Sydney, Sydney, NSW, Australia; 7 Faculty of Medicine and Health, The University of Sydney, Sydney, NSW, Australia; 8 The Woolcock Institute for Medical Research, Glebe, NSW, Australia

**Keywords:** Bayesian modelling, space-time interaction, spatio-temporal analysis, Tuberculosis, Vietnam

## Abstract

Spatial analysis and disease mapping have the potential to enhance understanding of tuberculosis (TB) dynamics, whose spatial dynamics may be complicated by the mix of short and long-range transmission and long latency periods. TB notifications in Nam Dinh Province for individuals aged 15 and older from 2013 to 2022 were analyzed with a variety of spatio-temporal methods. The study commenced with an analysis of spatial autocorrelation to identify clustering patterns, followed by the evaluation of several candidate Bayesian spatio-temporal models. These models varied from simple assessments of spatial heterogeneity to more complex configurations incorporating covariates and interactions. The findings highlighted a peak in the TB notification rate in 2017, with 98 cases per 100,000 population, followed by a sharp decline in 2021. Significant spatial autocorrelation at the commune level was detected over most of the 10-year period. The Bayesian model that best balanced goodness-of-fit and complexity indicated that TB trends were associated with poverty: each percentage point increase in the proportion of poor households was associated with a 1.3% increase in TB notifications, emphasizing a significant socioeconomic factor in TB transmission dynamics. The integration of local socioeconomic data with spatio-temporal analysis could further enhance our understanding of TB epidemiology.

## Introduction

Despite recent advances, tuberculosis (TB), the infectious disease caused by *Mycobacterium tuberculosis* (*M.tb*), continues to pose substantial public health hurdles due to diagnostic challenges, treatment complexities and the emergence of drug-resistant strains [1]. Vietnam, recognized as one of the top 30 countries for TB burden, exemplifies the global struggle against this infectious threat [2].

As a directly transmitted infectious disease, TB is linked with several population characteristics that may be spatially correlated, although there is a variable and often lengthy delay between infection and disease onset [3]. As such, people living in high-burden areas might actively spread TB to neighbouring regions [4]. While TB notifications do not directly reflect the true underlying incidence of TB, they still provide valuable information about TB burden and the effectiveness of control measures. Notifications reflect the number of TB cases that have been identified and reported, which can be influenced by the underlying transmission of *M.tb*, as well as factors including novel diagnostic techniques, enhanced detection efforts and the overall treatment outcomes achieved by control programs [5, 6].

Spatial analysis and disease mapping are powerful methods for understanding infectious diseases such as TB. These techniques can identify high-incidence areas, improve understanding of transmission dynamics, evaluate the impact of public health interventions and investigate socioeconomic risk factors. These insights can support the design of targeted interventions, with the potential to allocate resources more efficiently and reduce the burden of TB [7].

Bayesian spatio-temporal analysis methods have gained popularity in disease mapping over recent decades. By incorporating prior knowledge and leveraging data from appropriate neighbouring areas or time points, these methods provide more stable and accurate estimates, even in regions with sparse data. Utilizing these approaches enhances our ability to discern patterns and relationships that might otherwise be obscured, offering the potential for insights into both the current state of the disease and its future course. This can prove invaluable in planning and implementing more effective public health strategies, optimizing intervention timing, and understanding the factors driving disease spread, ultimately leading to more informed decision-making and improved outcomes in TB control [8, 9].

Bayesian spatio-temporal modelling for diseases has progressed significantly over recent decades, starting from the foundational models of Clayton and Kaldor (1987) and Besag et al. (1991) [8]. Bernardinelli et al. (1995) added models with area-specific intercepts and assumed linear temporal trends [10]. Waller et al. (1997) extended this framework by treating time as exchangeable, using Besag et al.’s hierarchical model independently at each time point without considering temporal risk. Knorr-Held and Besag (1998) merged this spatial model with dynamic, non-parametric models for estimating temporal trends, keeping temporal and spatial effects additive. Knorr-Held further developed these models, proposing a framework of four types of space-time interactions that explain disease variation beyond simple temporal and spatial effects. These range from unstructured (Type I) to complex structured interactions of space and time (Type IV) [10, 11].

In this study, we applied Bayesian approaches to investigate the spatial and temporal distribution of routinely notified TB cases during the period 2013 to 2022 and identified potential influencing factors at the local level in a single province in Vietnam.

We selected Nam Dinh Province for our analysis because its data is considered reliable by national authorities, and TB notification rates are similar to the national average. Its combination of urban and rural populations, and diverse socio-economic conditions make it a useful case study for understanding *M.tb* transmission dynamics, with findings potentially applicable to the broader national context of Vietnam and beyond.

## Methods

### Settings

Nam Dinh Province is located in the southern part of Vietnam’s Red River Delta, covers 1,676 km^2^ and has a population of approximately 1.83 million. The population is spread over one provincial city, which consists of 22 wards and three communes, and nine rural districts, comprising 201 towns/communes. Characterized by flat terrain, the province has higher population densities in the provincial city (Nam Dinh City), reflecting the typical urban concentration of a regional economic and administrative hub.

### Datasets

**Figure 1.**
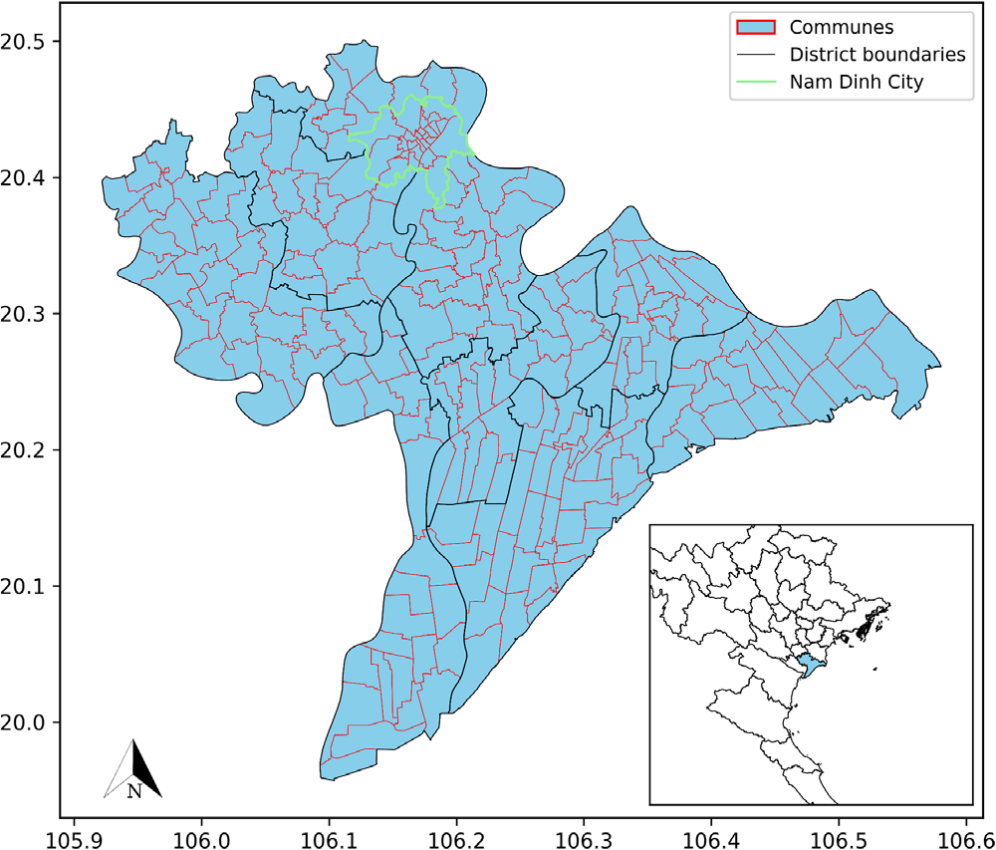
Base map of Nam Dinh Province. *World Geodetic System 84 Universal Transverse Mercator Zone 48N.*

We analyzed TB notifications in Nam Dinh Province from 2013 to 2022, considering all forms of TB (new and relapsed cases, both pulmonary and extrapulmonary TB and including non-bacteriologically confirmed cases) occurring in individuals aged 15 and above. Notification data, aggregated at the commune level, was sourced from the Vietnam National TB Programme’s database. Additionally, we acquired a dataset at the commune level from the Nam Dinh Statistical Office, detailing the names of communes, population data and proportion of households living below the national poverty standard over the analysis period, henceforward proportion of poor households. Map figures were produced using a base map of Vietnam from the Database of Global Administrative Areas [12], including commune names, administrative codes, and land areas for visualization (Figure 1).

### Data analysis

#### Utilization of the adjacency matrix

In spatial analysis, the adjacency matrix, which defines border relationships between areal units, is a commonly used basis for conducting spatial autocorrelation and modelling. To define spatial relationships among areas, we utilized the queen contiguity matrix [13], such that two communes were considered neighbours if they shared at least one vertex. Figure S1 shows the spatial relationships among 226 communes through the queen contiguity matrix.

#### Spatial autocorrelation analysis

The expected number of TB cases for each area *i* in year *t*, denoted 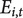 , was calculated using the formula:

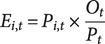

where:

- 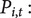 denotes the population in area *i* at year *t*,

- 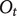 : denotes the total notified TB cases of all communes for year *t*,

- 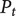 : denotes the total population of all communes for year *t*,

- *i* indexes spatial patches (communes) from 1 to 226,

- *t* indexes study years from 2013 to 2022.

The spatial standardized morbidity risk (SMR) for each commune *i* at year *t* is calculated as:

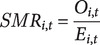

where 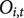 denotes the notified TB cases in commune *i* at year *t.*

The 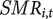 indicates whether a commune has a higher ( 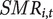 > 1) or lower ( 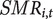 < 1) risk than would be expected from the whole province at that specific time point.

We analyzed spatial patterns with the Global Moran’s I Statistic [14], which evaluates overall spatial autocorrelation to identify clustering of TB SMRs.

Details of the calculations of Moran’s I are described in the Supplementary Material.

#### Bayesian spatio-temporal modelling

Our Bayesian spatio-temporal models assumed that the number of cases observed in commune *i* and year *t* followed a Poisson distribution:

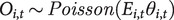

Where 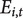 is the expected number of cases, and 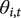 is the SMR of commune *i* and year *t.*

We considered 12 candidate models, each addressing various aspects of TB dynamics, ranging from basic spatial distributions to complex interactions over both time and space. Each base model, labelled from Model 1a to Model 6a, was replicated with the inclusion of areal covariates, producing corresponding versions Model 1b through Model 6b.

Model 1a – Besag-York-Mollié (BYM) model: This model integrates both structured and unstructured spatial effects [15]. The structured spatial effect follows the conditional autoregressive (CAR) approach, where the estimate for each commune is influenced by those of neighbouring communes through the queen contiguity matrix introduced above. In contrast, the unstructured spatial effect aims to capture independent random noise across distinct locations. There is no time structure nor time component in this model, meaning that observations from different years are treated as independent data points for each spatial unit, without accounting for temporal correlation or trends.

Next, we considered models that included temporal structure. Initial analysis showed that the trend of TB SMR of communes during the study period was not linear, as reflected in the decline in TB notifications in 2021. As a result, we did not pursue models with a linear time trend and instead focused on models incorporating a random walk over time. This statistical method models each time period’s estimates as updating the previous period’s values, allowing for random but dependent changes over time.

Model 2a: This model included unstructured spatial effects [15] and a temporal random walk. No spatial dependency between neighbouring communes was assumed.

Model 3a **– BYM model with random walk in time:** We retained the spatial framework of the Model 1a (the BYM model) while incorporating a random walk to account for time-dependent changes in SMRs [15].

Next, we explored several types of space-time interaction models [10, 11, 15].

Model 4a **– BYM model with a temporal random walk and Knorr-Held type I space-time interactions:** This model used the BYM model and a temporal random walk, combined with unstructured interactions for both time and space. This allowed the model to capture random fluctuations in TB SMR across different periods and locations, accounting for unstructured variability in both dimensions [10].

Model 5a **– BYM model with temporal random walk and Knorr-Held type II space-time interactions:** In this model, structured temporal effects interact with unstructured spatial effects. This helps to capture random spatial fluctuations and regular temporal trends in TB SMRs [10].

Model 6a **– BYM model with temporal random walk and Knorr-Held type III space-time interactions:** This model captures the interaction of unstructured temporal effects with structured spatial effects, enabling it to account for both random temporal fluctuations and organized spatial trends or gradients [10].

During the preliminary analysis, we investigated the possibility of Type IV interactions [10] to allow for structured interactions across both space and time. However, due to its high number of parameters and the consequent difficulty in fitting this model, we focused on more manageable models that could consistently capture the key relationships within the data.

#### Selection of priors

In Bayesian disease mapping, hyperparameters play an important role in shaping the prior distributions of model parameters. These hyperparameters influence the degree of regularization and the flexibility of the model, guiding parameter estimation based on prior knowledge [16]. For spatial effects, these include precision parameters for structured effects, and for unstructured random noise, which determine the degree of spatial smoothness and variability. For temporal effects, precision parameters in random walk priors control the strength of temporal dependency and the flexibility to capture trends over time. These hyperparameters guide parameter estimation by incorporating prior knowledge, ensuring accurate modelling of spatiotemporal patterns in disease risk [11, 17]. Initially, all proposed models were fitted using a log-gamma distribution as the prior for the hyperparameters governing the effect of the spatial and temporal variables, with a shape parameter of 1 and a rate of 0.0005. This choice reflects a weakly informative prior, providing broad flexibility to the model.

#### Covariate selection

Given the availability of relevant data, we selected the commune-level population density and proportion of households living below the national poverty line [18] as candidate covariates closely related to crowding and poorly ventilated living conditions. As such, we considered them as independent variables that may each influence *M.tb* transmission. Specifically, population density may affect the likelihood of *M.tb* spreading due to close contact, while poverty may impact access to healthcare and living conditions, both of which may be important to *M.tb* transmission dynamics [19]. Table S1 provides summary statistics for the covariates used in the analysis. The proportion of poor households across communes has a mean of 3.50%, with a standard deviation (SD) of 2.80% and an interquartile range (IQR) from 1.44% to 5.90%. The average population density was 2.5 thousand people/km², with an SD of 2.50 and an IQR ranging from 0.8 to 1.41 thousand people/km².

Details of the Bayesian spatio-temporal modeling are provided in the Supplementary Material.

#### Model evaluation

Model performance was evaluated using the mean posterior deviance, Deviance Information Criterion (DIC) [20] and Watanabe-Akaike Information Criterion (WAIC) [21]. While mean posterior deviance specifically assesses the model’s fit to the data, lower values of DIC and WAIC signify a model’s superior ability to balance goodness-of-fit with complexity, thereby indicating better overall model performance.

#### Sensitivity *analysis*

In the initial analysis, we modelled the counts of TB notifications using a Poisson distribution. While the analyzed model accounted for random spatial effects of TB notifications, we also explored a negative binomial distribution to assess the model’s sensitivity to the statistical distribution chosen to capture the case count data.

To assess the robustness of the best-fitting model, we conducted sensitivity analyses by testing alternative sets of hyperparameters for the log-gamma distribution. Specifically, we used a shape parameter of 0.01 with a rate of 0.01, representing a highly diffuse prior, and a shape parameter of 2 with a rate of 0.5, reflecting a more concentrated prior. These variations allowed us to evaluate how sensitive the model results were to changes in our prior assumptions. Figure S2 (Supplementary Material) shows the distribution of log-gamma distribution with different values of shape and rate.

#### 3.3. Software

We used R 4.3.3 [22] for data analysis and visualization. R-INLA [17] was used for spatio-temporal modelling.

## Results

**Figure 2.**
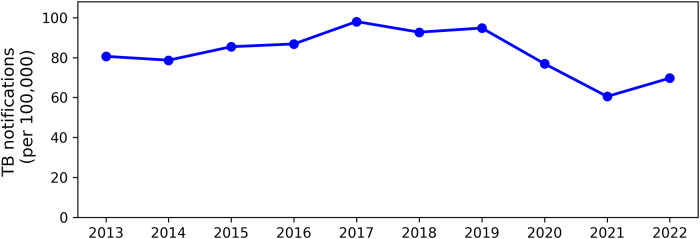
TB notifications per 100,000 population in Nam Dinh Province by study year.

From 2013 to 2022, Nam Dinh Province reported a total of 14,887 TB cases in individuals aged over 15. Throughout this period, TB notification rates per 100,000 population fluctuated significantly, peaking in 2017 at approximately 98 cases per 100,000 population. By contrast, 2021 recorded the lowest rate at around 60 cases per 100,000 population. Figure 2 illustrates the notification rates from 2013 to 2022, while Figure S3 describes the number of cases notified by each commune.

### Spatial autocorrelation

Table 1 shows the Global Moran’s I statistics for each individual year within our study period. We observed clear but weak spatial autocorrelation in TB notified incidence throughout the study period. While Moran’s I values were modest, the autocorrelation appeared to weaken in later years.

**Table 1.** Global Moran’s I statistics

Year	Moran’s I	*p*-value
2013	0.094	<0.01**
2014	0.117	<0.01**
2015	0.081	0.02*
2016	0.091	0.01*
2017	0.071	<0.01**
2018	0.060	0.06*
2019	0.129	<0.01**
2020	0.069	0.04*
2021	0.043	0.12
2022	0.051	0.08

*Source*: Moran’s I expected value: 0.444. * p <0.05, ** p < 0.01.

**Table 2.** Goodness of fit comparison of models

Model	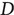	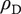	DIC	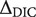	WAIC	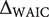
Model 1a	10507.05	165.26	10672.31	27.63	10696.48	35.58
Model 1b	10506.42	166.19	10672.61	27.93	10697.56	36.66
Model 2a	10517.63	158.34	10675.97	31.29	10698.42	37.52
Model 2b	10517.57	159.50	10677.07	32.29	10700.63	39.73
Model 3a	10508.15	167.18	10675.33	30.65	10700.48	39.58
Model 3b	10506.71	167.96	10674.67	29.99	10700.19	39.29
Model 4a	10264.22	382.31	10646.53	1.85	10660.50	−0.40
Model 4b	10264.66	380.06	10644.68	Referent	10660.90	Referent
Model 5a	10292.21	511.07	10803.28	158.60	10808.15	147.25
Model 5b	10235.32	601.75	10837.07	192.39	10820.21	159.31
Model 6a	10354.35	290.04	10644.38	−0.30	10667.24	6.34
Model 6b	10375.62	273.49	10649.11	4.43	10673.56	12.66

*Source*: D: Posterior mean deviance, 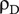 : Effective numbers of parameters, DIC: Deviance Information Criterion, 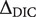 , 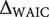 : Difference in DIC and WAIC relative to Model 4b, respectively. Our preferred model (Model 4b) was used as the referent analysis for comparison of information criteria.

### Bayesian spatio-temporal modelling

Table 2 presents a comparison of the candidate models’ goodness-of-fit metrics, including those incorporating the effective number of parameters. Though Models 4a and 4b were both potential candidates based on their DIC and WAIC values, Model 4b was our preferred choice for further analysis owing to its lower DIC. Models 2a and 2b also demonstrated relatively strong performance, with moderate DIC and WAIC values. Model 5b had the lowest posterior mean deviance but also the highest effective number of parameters, resulting in the highest DIC. Model M4b had the additional advantage of permitting estimation of the effects of areal covariates.

**Figure 3.**
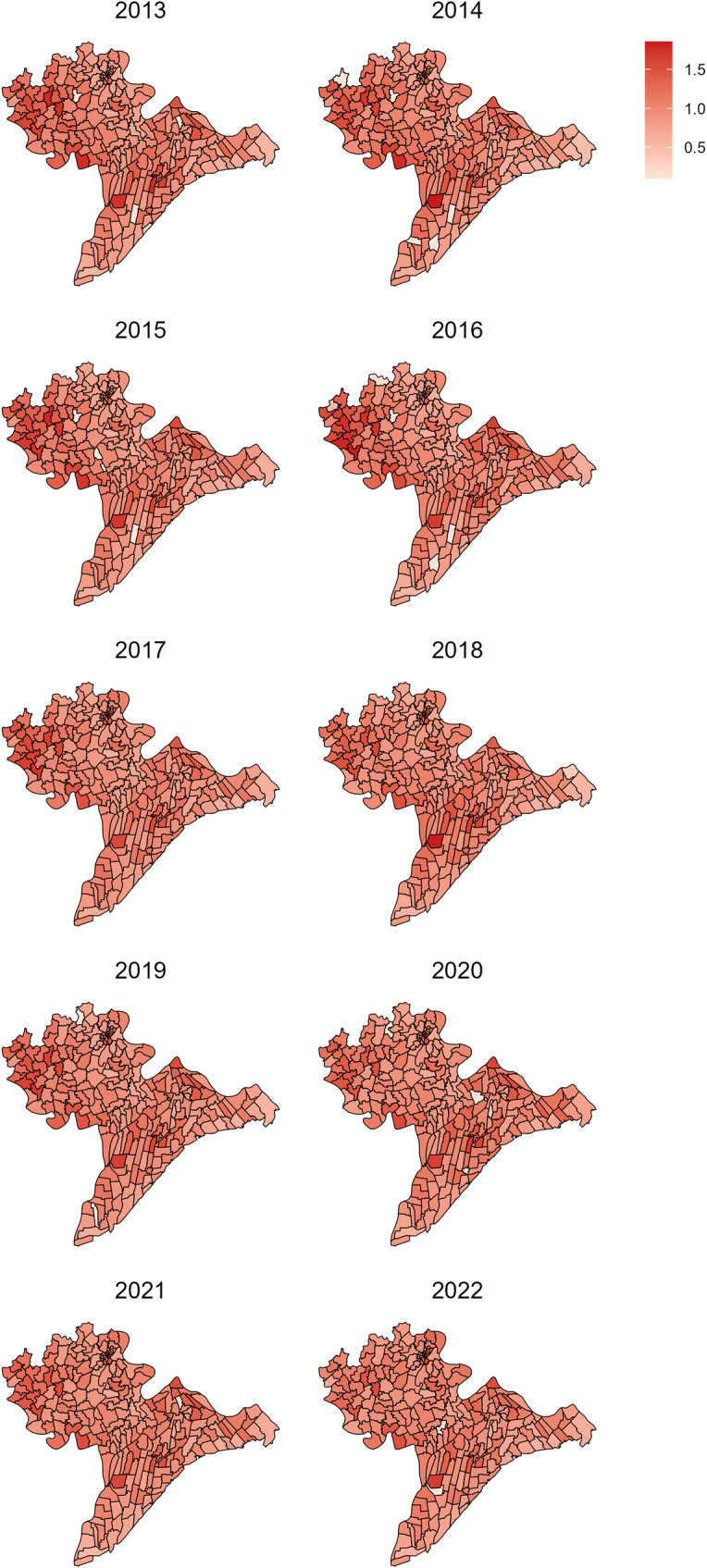
Predicted TB relative risks in Nam Dinh Province, from 2013 to 2022.

Table 3 presents the fixed effects of poor households and population density on TB SMRs using Model 4b. The proportion of poor households was significantly and positively correlated with TB SMRs. The distribution for the coefficient for poor household proportion indicated that each percentage point increase in the proportion of poor households of a commune was associated with a 1.3% (95% CrI: 0.1%–2.5%) increase in predicted TB notifications. On the other hand, population density did not appear to be associated with TB SMR. The mean and median estimation for the distribution of the coefficient was 0.001, indicating a 0.1% rise in TB notifications for each 1000 people/km^2^ change in population density. However, the 95% CrI (−0.009 to 0.01) indicates that it is plausible that population density had no association with TB notifications. Figure S4 (Supplementary Material) displays the marginal posterior distribution of the proportion of poor households, which represents the probability distribution of this parameter after accounting for the effects of all other parameters in the model. Figure 3 presents the model-predicted TB relative risks of 226 communes over the study period.

**Table 3.** Fixed effects of the model 4b

Fixed effect variable	Mean (SD)	Median (95% CrI)
Poor household proportion	0.013 (0.005)	0.013 (0.001–0.025)
Population density	0.001 (0.005)	0.001 (−0.009–0.01

*Source:* SD: standard deviation, 95% CrI: 0.025 quantile to 97.5 quantile.

### Sensitivity analysis

The choice of hyperpriors impacted the model’s performance, as reflected in differences in the effective number of parameters across prior choices (Table 4). Despite this, the estimates for key covariates remained qualitatively robust. For example, the fixed effect of poor household proportions showed minimal variations in posterior means and medians, with overlapping 95% CrIs across all prior configurations. Similarly, the fixed effect of population density demonstrated small changes, with consistent interpretations of the estimates.

**Table 4.** Sensitivity analysis results of the Model 4b

	Log-gamma prior		
	Shape = 1, Rate = 0.0005	Shape = 0.01, Rate = 0.01	Shape = 2, Rate = 0.5
Fixed effect of poor household proportion			
Mean (SD)	0.013 (0.005)	0.012 (0.007)	0.014 (0.007)
Median (95% CrI)	0.013 (0.001 to 0.025)	0.013 (0.001 to 0.025)	0.012 (0.001 to 0.025)
Fixed effect of population density			
Mean (SD)	0.001 (0.005)	0.001 (0.005)	−0.002 (0.005)
Median (95% CrI)	0.001 (−0.009 to 0.01)	0.001 (−0.009 to 0.01)	−0.002 (−0.011 to 0.002)

*Source*: 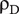 : Effective numbers of parameters, SD: standard deviation, 95% CrI: 0.025 quantile to 97.5 quantile.

Table S2 presents the goodness-of-fit comparison for models with a negative binomial distribution for TB notifications. Model 4b consistently exhibited the lowest DIC, achieving the best balance between the fit and complexity of the models considered.

## Discussion

We analyzed commune-level adult TB notification counts in Nam Dinh Province, Vietnam, from 2013 to 2022 at the smallest administrative level. Significant clustering was observed across 226 communes through spatial autocorrelation for most of the 10-year study period. Among 12 candidate Bayesian disease mapping models, those incorporating spatial and temporal effects along with unstructured interactions achieved the best balance between goodness-of-fit and complexity.

During this period, 14,887 TB cases were recorded, with the notification rate peaking at approximately 100 cases per 100,000 population in 2017 and falling to a low of about 60 cases per 100,000 in 2021. A likely contributor to the decline in TB notifications in 2021 was the COVID-19 pandemic, which disrupted TB control efforts through limited healthcare access, resource reallocation and changes in social behaviours that may have impacted transmission patterns [23, 24].

Overall, models that incorporated different types of space-time interactions (Models 4a to 6b) outperformed those with only spatial main effects (Model 1a and Model 1b), spatial unstructured effects and temporal effects (Model 2a and Model 2b), or a combination of spatial and temporal main effects (Model 3a and Model 3b). Among the models incorporating space-time interactions, Model 5b, which allowed for unstructured spatial and structured temporal effects with covariates, showed potential in capturing local dynamics and achieved the lowest posterior mean deviance. However, this model also exhibited a higher effective number of parameters, likely due to the complex temporal structure of the data. For example, some communes showed recent spikes in TB transmission with consistent increases in notifications, while others exhibited stable or declining trends. This variation could be attributable to the impact of recent transmission (as opposed to reactivation of latent TB infection), given the potentially long duration from infection to active disease. However, this interpretation is not conclusive because this approach did not achieve the best overall performance.

Notably, models incorporating spatial effects, temporal effects, and the interaction of unstructured spatial with temporal effects (Model 4a and Model 4b) showed the optimal balance between goodness-of-fit and complexity based on DIC values. This suggests that the spatial and temporal trends of TB may be influenced by neighbouring areas in the short term, with local outbreaks potentially spreading across adjacent regions, since only a small proportion of TB cases are likely to result from transmission between household members and known social contacts [25].

Incorporating the proportion of poor households and population density as covariates into Model 4b and 5b lowered the deviance. Although not a definitive finding, these results are consistent with other research that has found that social factors can contribute to variation in TB rates, particularly indicators of socioeconomic disadvantages [19, 26].

Our findings might have some limitations. First, the results may be sensitive to the modifiable area unit problem, which arises in spatial analysis when the results of a study are influenced by the choice of spatial units or boundaries used to aggregate the data [27]. *M.tb* transmission frequently occurs at the household level [28], leading to highly localized outbreaks. Our areal units, such as communes, may smooth over numerous individual epidemics occurring simultaneously within different households or communities. However, *M.tb* can also spread across large distances due to human mobility, driven by its prolonged disease duration and extended latency period [28]. This combination of very local transmission and long-range spread introduces unique challenges for spatial analysis of TB. Second, our analysis is based on commune-level data from the electronic system of the NTP, which, while being the best available data source, may be subject to reporting inaccuracies, incomplete case capture, or delays in notification. Such issues could introduce biases into the spatial patterns and temporal trends observed in our study. Additionally, this data may lack granularity on socioeconomic variables, comorbidities, or healthcare access, which could provide a more comprehensive understanding of the factors driving *M.tb* transmission.

Our systematic approach to model selection considered space, time, their interaction and socioeconomic factors over a significant period. By employing techniques such as spatial autocorrelation analysis, exploratory data visualization, and advanced Bayesian models, we uncovered TB patterns and trends that are not immediately apparent when only considering case counts in each area. By identifying communes with TB spikes or high-risk areas – such as those with elevated TB risk or higher poverty levels – resources can be allocated more effectively, prioritizing TB active case finding (ACF) activities in those areas. Studies have shown that combining spatial analysis with ACF can significantly increase TB detection in these areas [29]. However, this consideration must be balanced against the efficiency of applying policies and programs consistently across the country or individual provinces, which may be more efficient given the unpredictable nature of the epidemic. Additionally, integrating local socioeconomic data – such as income levels, education, population density, and healthcare access – into the TB surveillance system and applying Bayesian spatio-temporal analysis methods across broader regions could enhance predictive capabilities.

## Conclusions

We analyzed TB notifications in Nam Dinh Province from 2013 to 2022, finding clear but weak spatial autocorrelation across 226 communes over most of the 10-year period. Among the Bayesian methods evaluated, the model that incorporated spatial effects, temporal effects and unstructured interactions struck the best balance between complexity and goodness-of-fit. A more highly parameterized model with structured temporal and unstructured spatial interactions potentially offered the best fit to the evolving patterns of the data, suggesting that individual communes may exhibit distinct trends. Identifying consistent patterns without overfitting is challenging, likely due to the epidemic’s complexity and interacting epidemiological factors that include recent transmission, case detection, and past infection reactivation. The incorporation of socioeconomic indicators provided deeper insights into TB patterns, with the proportion of poor households identified as a likely influencing factor. This approach can be adapted to enhance TB management in Vietnam and other regions. Integrating areal socioeconomic data and other covariates with Bayesian spatio-temporal analysis across broader regions could enhance our understanding of TB epidemiology while collecting additional individual-level data through enhanced surveillance would be even more beneficial.

## Supporting information

Bui et al. supplementary material Bui et al. supplementary material

## Data Availability

The dataset includes sensitive individual information and cannot be publicly shared. Interested researchers should contact Dr. Nguyen Binh Hoa at nguyenbinhhoatb@yahoo.com. The code used for analysis is available on GitHub at github.com/longbui189/SPTB-VN.

## Supplementary material

For supplementary material accompanying this paper visit http://doi.org/10.1017/S0950268825000214. click here to view supplementary material
