# Supplementary material for: Bayesian spatio-temporal modelling of tuberculosis in Vietnam: Insights from a local-area analysis
Source: Epidemiol Infect. 2025 Feb 12;153:e34. doi: 10.1017/S0950268825000214 (PMC11869078; doi:10.1017/S0950268825000214)
Supplement: Bui et al. supplementary material [file S0950268825000214sup001.docx]

**BAYESIAN SPATIO-TEMPORAL MODELLING OF TUBERCULOSIS IN VIETNAM: INSIGHTS FROM A LOCAL-AREA ANALYSIS**

Viet Long Bui, Romain Ragonnet, Angus Hughes, Hoa Nguyen Binh^4^, Nam Do Hoang, Emma S. McBryde, Justin Sexton, Thuy Phuong Nguyen, David Shipman, Greg J. Fox, James M. Trauer

**SUPPLEMENTARY MATERIAL**

**Table of** **contents**

[I. METHODS 2](#_Toc188360859)

[1. Spatial Autocorrelation 2](#_Toc188360860)

[1.1. Expected TB Cases Calculation 2](#_Toc188360861)

[1.2. Global Moran’s I Calculation: 2](#_Toc188360862)

[2. Bayesian spatio-temporal modelling 4](#_Toc188360863)

[II. ADDITIONAL RESULTS: 6](#_Toc188360864)

[REFERENCES: 9](#_Toc188360865)

**List of Figures**

[Figure S1. Queen contiguity matrix of 226 communes of Nam Dinh Province 6](#_Toc185255282)

[Figure S2. Notified TB cases by commune in Nam Dinh Province, from 2023 to 2022... 6](#_Toc185255283)

[Figure S3. Distribution of different set of priors of the Model 4b. 7](#_Toc185255284)

[Figure S4. Marginal posterior distribution of poor household percentage with different priors in the Model 4b. 8](#_Toc185255285)

# I. METHODS

## 1. Spatial Autocorrelation

Spatial autocorrelation is a statistical measure that evaluates the degree to which similar values are located near each other in geographic space. It helps us understand if high or low values of a variable cluster together in a pattern that is different from what would be expected if the underlying spatial processes were random [1].

### 1.1. Expected TB Cases Calculation

The expected number of notified tuberculosis (TB) cases in each commune is calculated assuming that the overall notified TB cases across a province apply uniformly to each commune, weighted by the population of each commune.

The expected TB cases for each area *i* in a year *t*, denoted as $E_{i,t}$, can be calculated using the following formula:

$$E_{i,t}=P_{i,t}\times\frac{O_{t}}{P_{t}}$$

Where:

- $P_{i,t}:$the population in area *i* in year *t*,

- $O_{t}$: the total notified TB cases for all communes in year *t*,

- $P_{t}$: the total population of all communes in year *t*,

- *i* ranges from 1 to 226

- *t* ranges from 2013 to 2022, sequentially indexing the years of the study.

**The spatial Standardized Morbidity Risk (SMR) is calculated as:**

$$SMR_{i,t}=\frac{O_{i,t}}{E_{i,t}}$$

### 1.2. Global Moran’s I Calculation:

Global Moran's I is a measure of spatial autocorrelation that assesses whether the pattern of a variable across different spatial units (e.g., regions, districts) is clustered, dispersed, or random [2]. It compares the value of the variable at one location with values at neighbouring locations. A positive Moran's I indicates clustering of similar values, while a negative value indicates dispersion. The statistic is typically accompanied by a p-value to quantify the probability that of the observed spatial pattern arose through random chance alone.

Global Moran’s I is calculated as:

$$I=\frac{N}{W}\sum_{i=1}^{N} \sum_{j=1}^{N} w_{ij}\frac{\left( {SMR}_{i}- \overline{SMR} \right)\left( {SMR}_{j}-\overline{SMR} \right)}{\sum_{i=1}^{N} \left( {SMR}_{i}- \overline{SMR} \right)^{2}}$$

Where:

- *N* is the number of spatial units indexed by *i*,

- ${SMR}_{i}$ and $SMRj$ are the SMR values at communes *i* and *j*,

- $\overline{SMR}$ is the mean of SMR,

- $w_{ij}$ is the spatial weight between locations *i* and *j*

- W is the sum of all spatial weights

$$\sum_{i=1}^{N} \sum_{j=1}^{N} w_{ij}$$

A positive Moran’s I value indicates that communes with similar TB rates are clustered together, while a negative value suggests that high and low rates are interspersed, indicating dispersion.

#### 1.2.1. Calculating the p-value under normality assumption

**Formulate the hypotheses**

**- H₀**: There is no spatial autocorrelation.

**- H₁**: There is spatial autocorrelation.

**Compute the expected value and variance of Moran’s I**

Under the null hypothesis of no spatial autocorrelation, the expected value $E\left[ I \right]$ of Moran’s I is:

$$E\left[ I \right]=-\frac{1}{N-1}$$

The variance of Moran’s I $Var\left[ I \right]$under the normality assumption is given by:

$$Var\left[ I \right]=\frac{N^{2}S_{1}-NS_{2}+3W^{2}}{\left( N-1 \right)\left( N-2 \right)\left( N-3 \right)W^{2}}- {E[I]}^{2}$$

*where:*

$$S_{1}=\frac{1}{2}\sum_{i=1}^{N} \sum_{j=1}^{N} \left( w_{ij}+w_{ji} \right)^{2}$$

$$S_{2}=\sum_{i=1}^{N} \left( \sum_{j=1}^{N} w_{ij}+\sum_{j=1}^{N} w_{ji} \right)^{2}$$

**Standardize Moran’s I**

Standardize Moran’s I to convert it into a z-score:

$$z=\frac{I-E\left[ I \right]}{\sqrt{Var\left[ I \right]}}$$

**Determine the p-value**

Use the standard normal distribution to find the p-value corresponding to the calculated z-score. The p-value indicates the probability of observing a value as extreme as Moran’s I under the null hypothesis.

**Two-tailed test**: For a two-tailed test, the p-value is found as:

$$p=2\times\left( 1-\Phi\left( \left| z \right| \right) \right)$$

*Where* $\left( \Phi\right)$ is the cumulative distribution function (CDF) of the standard normal distribution.

**- p-value < 0.05**: Indicates that the observed spatial pattern is statistically significant, meaning there is less than a 5% likelihood of the pattern occurring through random chance. This suggests that clustering or dispersion is a real feature of the data.

**- p-value >= 0.05**: Suggests that the spatial pattern could be random, meaning the observed clustering or dispersion might not be statistically significant.

## 2. Bayesian spatio-temporal modelling

The Bayesian spatio-temoporal modelling model assumes that the number of cases $O_{i,j}$ observed in commune *i* and year *t* follows the Poisson distribution:

$$O_{i,t}\sim Poisson\left( E_{i,j}\theta_{i,t} \right)$$

$E_{i,j}$ is the expected number of cases, and $\theta_{i,j}$ is the SMR of commune *i* and year *t*.

In this study, we used a range of models to investigate the dynamics of *M.tb* transmission. These models aim to account for various spatial and temporal factors and include key covariates that affect TB incidence. The formulas for the models used in our study are described below:

**Model 1: Besag-York-Mollié (BYM) model**

| Model 1a (without covariate): | $log\left( \theta_{i,t} \right)=\alpha+u_{i} +v_{i}$ |
| --- | --- |
| Model 1b: (with covariates) | $log\left( \theta_{i,t} \right)=\alpha+u_{i} +v_{i}+\beta_{1}P_{i,t}+\beta_{2}D_{i,t}$ |

**Model 2: Unstructured spatial effects with random walk in time**

| Model 2a (without covariate): | $log\left( \theta_{i,t} \right)=\alpha+v_{i} + \gamma_{t}$ |
| --- | --- |
| Model 2b: (with covariates) | $log\left( \theta_{i,t} \right)=\alpha+v_{i} + \gamma_{t}+\beta_{1}P_{i,t}+\beta_{2}D_{i,t}$ |

**Model 3: BYM model with random walk in time**

| Model 3a (without covariate): | $log\left( \theta_{i,t} \right)=\alpha+u_{i} +v_{i}+\gamma_{t}$ |
| --- | --- |
| Model 3b (with covariates): | $log\left( \theta_{i,t} \right)=\alpha+u_{i} +v_{i}+\gamma_{t}+\beta_{1}P_{i,t}+\beta_{2}D_{i,t}$ |

*where:*

*-* $\alpha$: is the global intercept,

- $u_{i}$: represents the spatially structured effect in our Bayesian spatio-temporal models, utilizing the conditional autoregressive (CAR) framework of Besag [3]. It accounts for spatial correlation among observations by reflecting the influence of geographical proximity on TB rates across areas. The Besag model allows $u_{i}$ ​to be influenced by the average effects of neighboring areas.

- $v_{i}$: unstructured spatial heterogeneity in area *i*, accounts for the random variation in TB rates not explained by observed covariates or the spatial structure modeled by $u_{i}$. It captures effects from unmeasured variables or random events [3],

The $u_{i}$ + $v_{i}$ represents the combination of structured and unstructured spatial effects for each area in the Besag-York-Mollié (BYM) model [3].

- $P_{i,t}$ and $D_{i,t}$ are the covariates for the percentage of poor households and population density, respectively, in area *i* at time 𝑡*.* The corresponding coefficients for these covariates are with $\beta_{1}$ for $P_{i,t}$ and $\beta_{2}$ for $D_{i,t}$

- $\gamma_{t}$ (in Model 2): In a random walk of order 2, the value at time *t*, $\gamma_{t}$, depends on the values from the two previous time points, $\gamma_{t-1}$​ and $\gamma_{t-2}$​. The idea is that the difference from one time point to the next is influenced by the changes that occurred in the preceding steps, making the path of $\gamma_{t}$​ more stable and smoother. This model assumes that any sudden changes are normally distributed, meaning they follow a pattern that is typical and predictable over time [4].

From Model 3 to Model 5, the specifications follow different types of space-time interactions proposed by Knorr-Held [4,5].

**Model 4 to Model 6: BYM model with temporal random walk and space-time interactions**

| Without covariates: | $log\left( \theta_{i,t} \right)=\alpha+u_{i} +v_{i}+\gamma_{t} +\Phi_{t}+\delta_{it}$ |
| --- | --- |
| With covariates: | $log\left( \theta_{i,t} \right)=\alpha+u_{i} +v_{i}+\gamma_{t}+\Phi_{t}+ \delta_{it}+\beta_{1}P_{i,t}+\beta_{2}D_{i,t}$ |

Where $\Phi_{t}$ is unstructured temporal effect, $\delta$ represents different types of interaction space-time random effect.

| **Model** | **Knorr-Held’s Interaction type** | **Parameters interacting** |
| --- | --- | --- |
| Model 4a and Model 4b | I | $v_{i}$ and $\Phi_{t}$ |
| Model 5a and Model 5b | II | $v_{i}$ and $\gamma_{t}$ |
| Model 6a and Model 6b | III | $u_{i}$ and $\Phi_{t}$ |

# II. ADDITIONAL RESULTS:

**
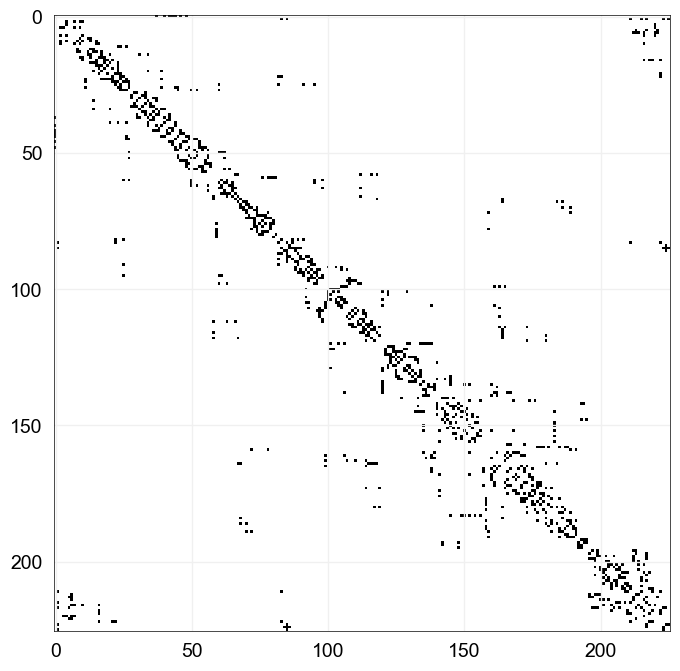
**

**Figure S1. Queen contiguity matrix of the 226 communes of Nam Dinh Province.**


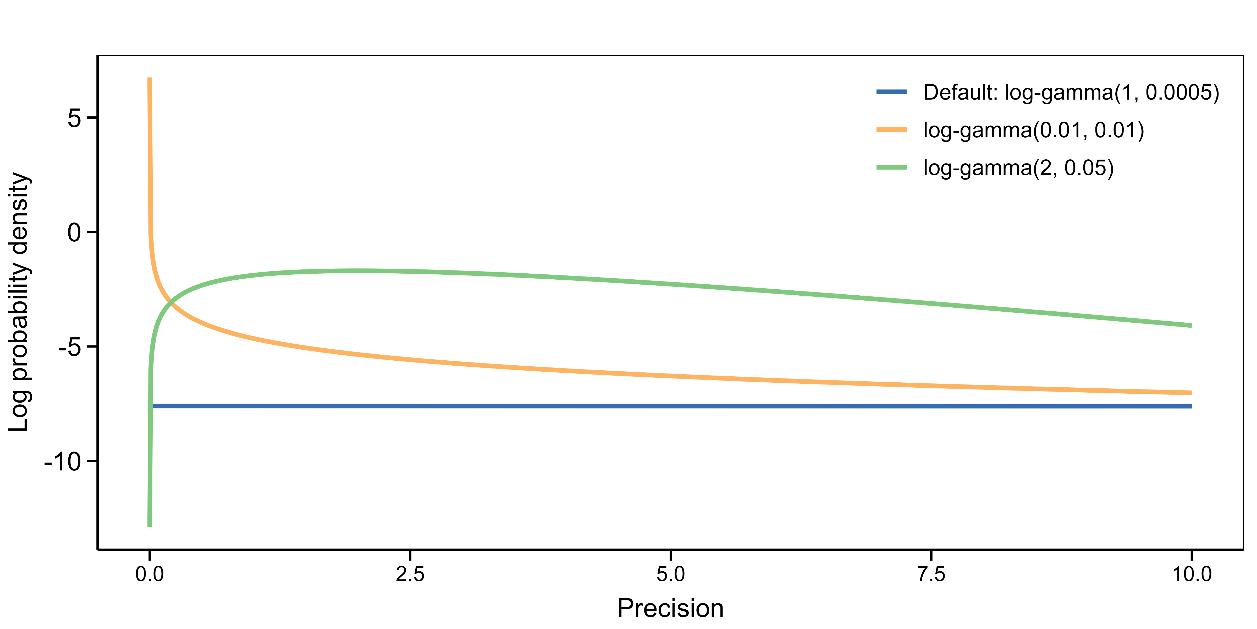


**Figure S2. Distribution of different set of priors of the Model 4b.**


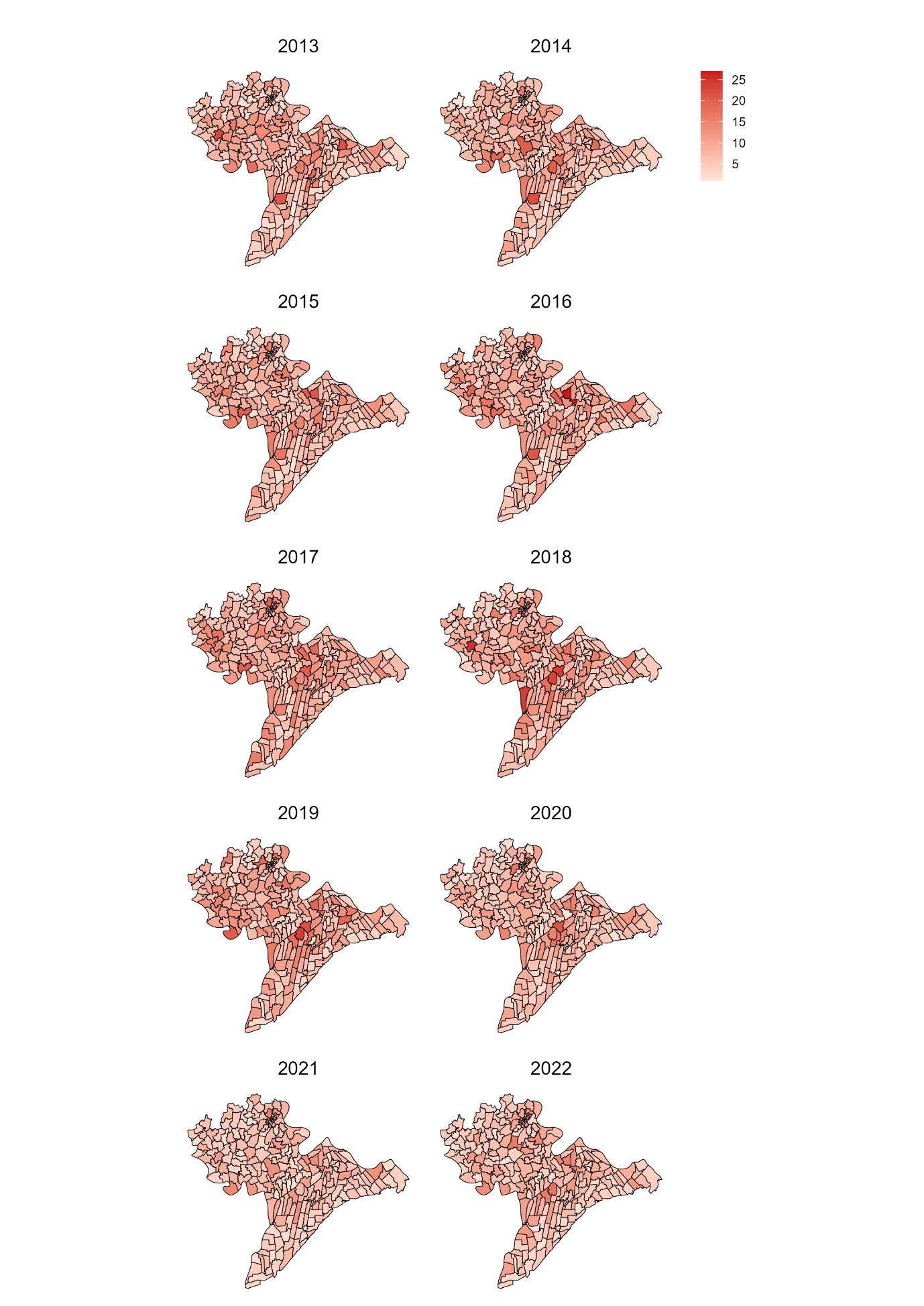


**Figure S3. Notified TB cases by commune in Nam Dinh Province, from 2023 to 2022.**


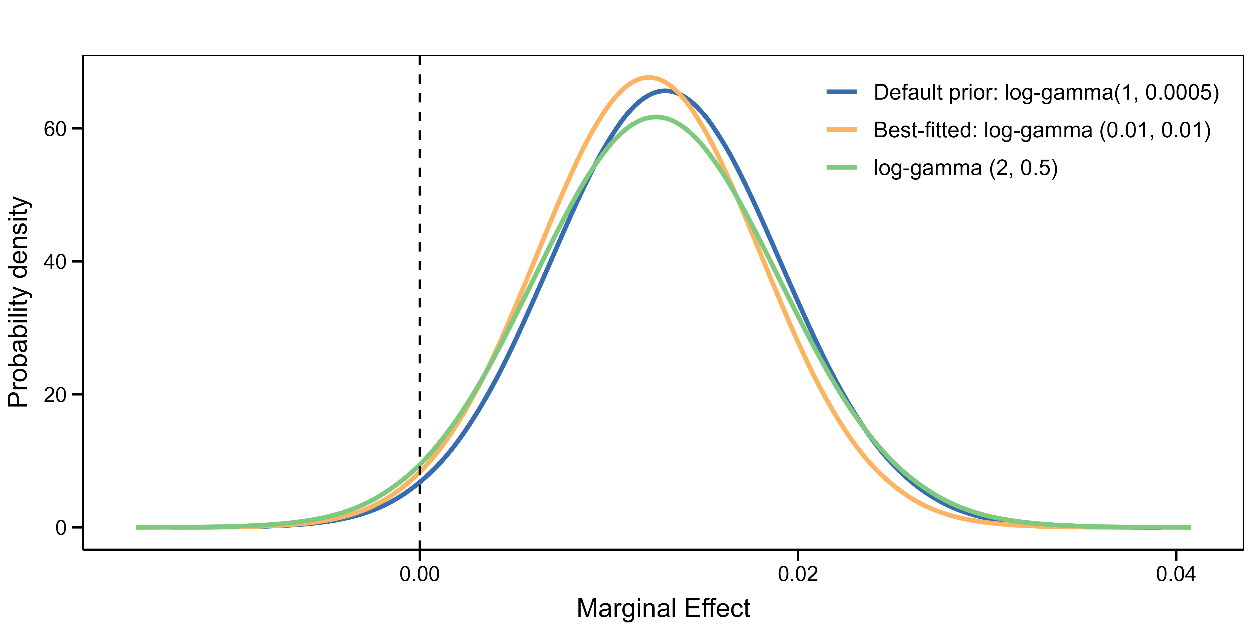


**Figure S4. Marginal posterior distribution of poor household percentage with different priors in the Model 4b.**

**Table S1. Summary statistics of c****ovariates**

| **Covariate** | **Mean** | **Standard deviation** | **Median** | **Interquartile range** |
| --- | --- | --- | --- | --- |
| Poor households’ proportion | 3.50 | 2.80 | 2.60 | 1.39 – 5.90 |
| Population density (thousand people/km²) | 2.45 | 2.50 | 1.19 | 0.84 – 1.41 |

**Table S2.** **Goodness-of-fit comparison of models with negative binomial distribution for TB notifications**

| **Model** | $\bar{\boldsymbol{D}}$ | $\boldsymbol{\rho}_{\mathbf{D}}$ | **DIC** | $\boldsymbol{\Delta}_{\mathbf{DIC}}$ | **WAIC** | $\boldsymbol{\Delta}_{\mathbf{WAIC}}$ |
| --- | --- | --- | --- | --- | --- | --- |
| **Model 1a** | 10520.67 | 169.6405 | 10690.31 | 15.11 | 10692.91 | 11.93 |
| **Model 1b** | 10520.85 | 169.4824 | 10690.33 | 15.13 | 10692.95 | 11.97 |
| **Model 2a** | 10517.64 | 158.3381 | 10682.97 | 7.77 | 10688.08 | 7.10 |
| **Model 2b** | 10517.57 | 159.5021 | 10687.07 | 11.87 | 10689.43 | 8.45 |
| **Model 3a** | 10519.68 | 159.2613 | 10688.94 | 13.74 | 10681.62 | 0.64 |
| **Model 3b** | 10518.51 | 160.4502 | 10678.96 | 3.76 | 10681.91 | 0.93 |
| **Model 4a** | 10517.18 | 161.2295 | 10678.41 | 3.21 | 10680.59 | -0.39 |
| **Model 4b** | 10516.74 | 162.4635 | 10675.20 | 0.00 | 10680.98 | 0.00 |
| **Model 5a** | 10271.04 | 544.5028 | 10815.54 | 140.34 | 10812.95 | 131.97 |
| **Model 5b** | 10320.08 | 511.8836 | 10831.96 | 156.76 | 10821.45 | 140.47 |
| **Model 6a** | 10413.72 | 240.6710 | 10704.39 | 29.19 | 10685.44 | 4.46 |
| **Model 6b** | 10502.67 | 212.5783 | 10715.24 | 40.04 | 10694.22 | 13.24 |

$\bar{D}$*: posterior mean deviance,* $\rho_{D}$*: Effective numbers of parameters, DIC: Deviance Information Criterion,* $\Delta_{DIC}$*,* $\Delta_{WAIC}$ *Difference in DIC and WAIC relative to Model 4b, respectively. Our preferred model (Model 4b) was used as the referent analysis for comparison of information criteria.*

# REFERENCES:

1. **Getis A**. A History of the Concept of Spatial Autocorrelation: A Geographer’s Perspective. *Geographical Analysis* 2008; **40**: 297–309.

2. **Moran PAP**. Notes on Continuous Stochastic Phenomena. *Biometrika* [Oxford University Press, Biometrika Trust], 1950; **37**: 17–23.

3. **Besag J, York J, Mollié A**. Bayesian image restoration, with two applications in spatial statistics. *Annals of the Institute of Statistical Mathematics* 1991; **43**: 1–20.

4. **Knorr-Held L**. Bayesian modelling of inseparable space-time variation in disease risk. *Statistics in Medicine* 2000; **19**: 2555–2567.

5. **Blangiardo M, *et al.*** Spatial and spatio-temporal models with R-INLA. *Spatial and Spatio-temporal Epidemiology* 2013; **4**: 33–49.
